# Determining sample size for progression criteria for pragmatic pilot RCTs: the hypothesis test strikes back!

**DOI:** 10.1186/s40814-021-00770-x

**Published:** 2021-02-03

**Authors:** M. Lewis, K. Bromley, C. J. Sutton, G. McCray, H. L. Myers, G. A. Lancaster

**Affiliations:** 1 Biostatistics Group, School of Medicine, Keele University, Room 1.111, David Weatherall Building, Keele, Staffordshire ST5 5BG UK; 2 Keele Clinical Trials Unit, Keele University, Keele, Staffordshire UK; 3 Centre for Biostatistics, School of Health Sciences, University of Manchester, Manchester, Staffordshire UK

**Keywords:** Outcome and process assessment, Pilots, Sample size, Statistics

## Abstract

**Background:**

The current CONSORT guidelines for reporting pilot trials do not recommend hypothesis testing of clinical outcomes on the basis that a pilot trial is under-powered to detect such differences and this is the aim of the main trial. It states that primary evaluation should focus on descriptive analysis of feasibility/process outcomes (e.g. recruitment, adherence, treatment fidelity). Whilst the argument for not testing clinical outcomes is justifiable, the same does not necessarily apply to feasibility/process outcomes, where differences may be large and detectable with small samples. Moreover, there remains much ambiguity around sample size for pilot trials.

**Methods:**

Many pilot trials adopt a ‘traffic light’ system for evaluating progression to the main trial determined by a set of criteria set up a priori. We construct a hypothesis testing approach for binary feasibility outcomes focused around this system that tests against being in the RED zone (unacceptable outcome) based on an expectation of being in the GREEN zone (acceptable outcome) and choose the sample size to give high power to reject being in the RED zone if the GREEN zone holds true. Pilot point estimates falling in the RED zone will be statistically non-significant and in the GREEN zone will be significant; the AMBER zone designates potentially acceptable outcome and statistical tests may be significant or non-significant.

**Results:**

For example, in relation to treatment fidelity, if we assume the upper boundary of the RED zone is 50% and the lower boundary of the GREEN zone is 75% (designating unacceptable and acceptable treatment fidelity, respectively), the sample size required for analysis given 90% power and one-sided 5% alpha would be around *n* = 34 (intervention group alone). Observed treatment fidelity in the range of 0–17 participants (0–50%) will fall into the RED zone and be statistically non-significant, 18–25 (51–74%) fall into AMBER and may or may not be significant and 26–34 (75–100%) fall into GREEN and will be significant indicating acceptable fidelity.

**Discussion:**

In general, several key process outcomes are assessed for progression to a main trial; a composite approach would require appraising the rules of progression across all these outcomes. This methodology provides a formal framework for hypothesis testing and sample size indication around process outcome evaluation for pilot RCTs.

**Supplementary Information:**

The online version contains supplementary material available at 10.1186/s40814-021-00770-x.

## Background

The importance and need for pilot and feasibility studies is clear: “A well-conducted pilot study, giving a clear list of aims and objectives … will encourage methodological rigour … and will lead to higher quality RCTs” [1]. The CONSORT extension to external pilot and feasibility trials was published in 2016 [2] with the following key methodological recommendations: (i) investigate areas of uncertainty about the future definitive RCT; (ii) ensure primary aims/objectives are about feasibility, which should guide the methodology used; (iii) include assessments to address the feasibility objectives which should be the main focus of data collection and analysis; and (iv) build decision processes into the pilot design whether or how to proceed to the main study. Given that many trials incur process problems during implementation—particularly with regard to recruitment [3–5]—the need for pilot and feasibility studies is evident.

One aspect of pilot and feasibility studies that remains unclear is the required sample size. There is no consensus but recommendations vary from 10 to 12 per group through to 60–75 per group depending on the main objective of the study. Sample size may be based on precision of a feasibility parameter [6, 7]; precision of a clinical parameter which may inform main trial sample size—particularly the standard deviation (SD) [8–11] but also event rate [12] and effect size [13, 14]; or, to a lesser degree, for clinical scale evaluation [9, 15]. Billingham et al. [16] reported that the median sample size of pilot and feasibility studies is around 30–36 per group but there is wide variation. Herbert et al. [17] reported that targets within internal as opposed to external pilots are often slightly larger and somewhat different, being based on percentages of the total sample size and timeline rather than any fixed sample requirement.

The need for a clear directive on sample size of studies is of upmost relevance. The CONSORT extension [2] reports that “Pilot size should be based on feasibility objectives and some rationale given” and states that a “confidence interval approach may be used to calculate and justify the sample size based on key feasibility objective(s)”. Specifically, item 7a (How sample size was determined: Rationale for numbers in the pilot trial) qualifies: “Many pilot trials have key objectives related to estimating rates of acceptance, recruitment, retention, or uptake … for these sorts of objectives, numbers required in the study should ideally be set to ensure a desired degree of precision around the estimated rate”. Item 7b (When applicable, explanation of any interim analyses and stopping guidelines) is generally an uncommon scenario for pilot and feasibility studies and is not given consideration here.

A key aspect of pilot and feasibility studies is to inform progression to the main trial, which has important implications for all key stakeholders (funders, researchers, clinicians and patients). The CONSORT extension [2] states that “decision processes about how to proceed needs to be built into the pilot design (which might involve formal progression criteria to decide whether to proceed, proceed with amendments, or not to proceed)” and authors should present “if applicable, the pre-specified criteria used to judge whether or how to proceed with a future definitive RCT; … implications for progression from pilot to future definitive RCT, including any proposed amendments”. Avery et al. [18] published recommendations for internal pilots emphasising a traffic light (stop-amend-go/red-amber-green) approach to progression with focus on process assessment (recruitment, protocol adherence, follow-up) and transparent reporting around the choice of trial design and the decision-making processes for stopping, amending or proceeding to a main trial. The review of Herbert et al. [17] reported that the use of progression criteria (including recruitment rate) and traffic light stop-amend-go as opposed to simple stop-go is increasing for internal pilot studies.

A common misuse of pilot and feasibility studies has been the application of hypothesis testing for clinical outcomes in small under-powered studies. Arain et al. [19] claimed that pilot studies were often poorly reported with inappropriate emphasis on hypothesis testing. They reviewed 54 pilot and feasibility studies published in 2007–2008, of which 81% incorporated hypothesis testing of clinical outcomes. Similarly, Leon et al. [20] stated that a pilot is not a hypothesis testing study: safety, efficacy and effectiveness should not be evaluated. Despite this, hypothesis testing has been commonly performed for clinical effectiveness/efficacy without reasonable justification. Horne et al. [21] reviewed 31 pilot trials published in physical therapy journals between 2012 and 2015 and found that only 4/31 (13%) carried out a valid sample size calculation on effectiveness/efficacy outcomes but 26/31 (84%) used hypothesis testing. Wilson et al. [22] acknowledged a number of statistical challenges in assessing potential efficacy of complex interventions in pilot and feasibility studies. The CONSORT extension [2] re-affirmed many researchers’ views that formal hypothesis testing for effectiveness/efficacy is not recommended in pilot/feasibility studies since they are under-powered to do so. Sim’s commentary [23] further contests such testing of clinical outcomes stating that treatment effects calculated from pilot or feasibility studies should not be the basis of a sample size calculation for a main trial.

However, when the focus of analysis is on confidence interval estimation for process outcomes, this does not give a definitive basis for acceptance/rejection of progression criteria linked to formal powering. The issue in this regard is that precision focuses on alpha (*α*, type I error) without clear consideration of beta (β, type II error) and may therefore not reasonably capture true differences if a study is under-powered. Further, it could be argued that hypothesis testing of feasibility outcomes (as well as addressing both alpha and beta) is justified on the grounds that moderate-to-large differences (‘process-effects’) may be expected rather than small differences that would require large sample numbers. Moore et al. [24] previously stated that some pilot studies require hypothesis testing to guide decisions about whether larger subsequent studies can be undertaken, giving the following example of how this could be done for feasibility outcomes: asking the question “Is taste of dietary supplement acceptable to at least 95% of the target population?”, they showed that sample sizes of 30, 50 and 70 provide 48%, 78% and 84% power to reject an acceptance rate of 85% or lower if the true acceptance rate is 95% using a 1-sided *α* = 0.05 binomial test. Schoenfeld [25] advocates that, even for clinical outcomes, there may be a place for testing at the level of clinical ‘indication’ rather than ‘clinical evidence’. He suggested that preliminary hypothesis testing for efficacy could be conducted with high alpha (up to 0.25), not to provide definitive evidence but as an indication as to whether a larger study should be conducted. Lee et al. [14] also reported how type 1 error levels other than the traditional 5% could be considered to provide preliminary evidence for efficacy, although they did stop short of recommending doing this by concluding that a confidence interval approach is preferable.

Current recommendations for sample sizes of pilot/feasibility studies vary, have a single rather than a multi-criterion basis, and do not necessarily link directly to formal progression criteria. The purpose of this article is to introduce a simple methodology that allows sample size derivation and formal testing of proposed progression cut-offs, whilst offering suggestions for multi-criterion assessment, thereby giving clear guidance and sign-posting for researchers embarking on a pilot/feasibility study to assess uncertainty in feasibility parameters prior to a main trial. The suggestions within the article do not directly apply to internal pilot studies built into the design of a main trial, but given the similarities to external randomised pilot and feasibility studies, many of the principles outlined here for external pilots might also extend to some degree to internal pilots of randomised and non-randomised studies.

## Methods

The proposed approach focuses on estimation and hypothesis testing of progression criteria for feasibility outcomes that are potentially modifiable (e.g. recruitment, treatment fidelity/ adherence, level of follow up). Thus, it aligns with the main aims and objectives of pilot and feasibility studies and with the progression stop-amend-go recommendations of Eldridge et al. [2] and Avery et al. [18].

### Hypothesis concept

Let *R*_*UL*_ denote the upper RED zone cut-off and *G*_*LL*_ denote the lower GREEN zone cut-off. The concept is to set up hypothesis testing around progression criteria that tests against being in the RED zone (designating unacceptable feasibility—‘*STOP*’) based on an alternative of being in the GREEN zone (designating acceptable feasibility—‘*GO*’). This is analogous to the zero difference (null) and clinically important difference (alternative) in a main superiority trial. Specifically, we are testing against *R*_*UL*_ when *G*_*LL*_ is hypothesised to be true:

- Null hypothesis: True feasibility outcome (*ε*) not greater than the upper “RED” *stop* limit (*R*_*UL*_)
- Alternative hypothesis: True feasibility outcome (*ε*) is greater than *R*_*UL*_

The test is a 1-tailed test with suggested alpha (*α*) of 0.05 and beta (β) of 0.05, 0.1 or 0.2, dependent on the required strength of evidence of the test. An example of a feasibility outcome might be percentage recruitment uptake.

### Progression rules

Let *E* denote the observed point estimate (ranging from 0 to 1 for proportions, or for percentages 0–100%). Simple 3-tiered progression criteria would follow as:

- *E* ≤ *R*_*UL*_ [*P* value non-significant (*P ≥ α*)] -> RED (unacceptable—STOP)
- *R*_*UL*_ < *E* < *G*_*LL*_ -> AMBER (potentially acceptable—AMEND)
- *E* ≥ *G*_*LL*_ [*P* value significant (*P < α*)] -> GREEN (acceptable—GO)

### Sample size

Table 1 displays a quick look-up grid for sample size across a range of anticipated proportions for *R*_*UL*_ and *G*_*LL*_ for one-sample one-sided 5% alpha with typical 80% and 90% (as well as 95%) power for the normal approximation method with continuity correction (see Appendix for corresponding mathematical expression; derived from Fleiss et al. [26]). Table 2 is the same look-up grid relating to the Binomial exact approach with sample sizes derived using G*Power version 3.1.9.7 [27]. Clearly, as the difference between proportions *R*_*UL*_ and *G*_*LL*_ increases the sample size requirement is reduced.

**Table 1:** Sample size and significance cut-points for (G_LL_-R_UL_) differences for a one-sample test, power (80%, 90%, 95%) and 1-tailed 5% significance level based on normal approximation (with continuity correction)

***R***_***UL***_	***G***_***LL***_	***α*** (0.05), ***β*** (0.2)		***α*** (0.05), ***β*** (0.1)		***α*** (0.05), ***β*** (0.05)	
%	%	***n***	***A***_***c***_ (%)	***n***	***A***_***c***_ (%)	***n***	***A***_***c***_ (%)
10	20	79	12.3 (15.6)	111	16.3 (14.7)	143	20.2 (14.1)
15	25	101	21.1 (20.8)	140	28.0 (20.0)	179	34.7 (19.4)
15	30	49	11.5 (23.4)	68	15.0 (22.1)	87	18.5 (21.3)
20	30	119	31.0 (26.0)	165	41.5 (25.1)	209	51.3 (24.6)
20	35	57	16.4 (28.7)	78	21.4 (27.5)	99	26.3 (26.6)
20	40	34	10.6 (31.3)	46	13.7 (29.7)	59	16.9 (28.6)
25	35	134	41.7 (31.2)	185	55.9 (30.2)	234	69.4 (29.7)
25	40	63	21.4 (34.0)	86	28.1 (32.7)	109	34.7 (31.8)
25	45	37	13.6 (36.7)	51	17.8 (35.0)	64	21.7 (33.9)
25	50	25	9.8 (39.2)	33	12.3 (37.4)	42	15.1 (36.0)
30	40	146	52.9 (36.2)	201	71.0 (35.3)	253	87.9 (34.7)
30	45	68	26.6 (39.1)	93	35.2 (37.8)	117	43.3 (37.0)
30	50	39	16.4 (42.1)	54	21.7 (40.3)	67	26.3 (39.2)
30	55	26	11.6 (44.8)	35	15.0 (42.7)	44	18.2 (41.4)
30	60	18	8.6 (47.8)	25	11.3 (45.1)	30	13.1 (43.8)
35	45	155	64.0 (41.3)	212	85.6 (40.4)	267	106.3 (39.8)
35	50	71	31.5 (44.3)	97	41.7 (43.0)	121	51.0 (42.1)
35	55	41	19.4 (47.3)	56	25.5 (45.5)	69	30.7 (44.4)
35	60	27	13.5 (50.1)	36	17.3 (48.1)	44	20.6 (46.8)
35	65	19	10.1 (53.0)	25	12.7 (50.7)	31	15.2 (49.1)
40	50	160	74.2 (46.4)	219	99.5 (45.4)	275	123.4 (44.9)
40	55	73	36.1 (49.4)	99	47.6 (48.1)	124	58.6 (47.2)
40	60	42	22.0 (52.4)	56	28.4 (50.8)	70	34.7 (49.6)
40	65	27	15.0 (55.5)	36	19.2 (53.4)	44	22.9 (52.1)
40	70	19	11.1 (58.5)	25	14.0 (56.1)	30	16.4 (54.7)
45	55	163	83.8 (51.4)	222	112.1 (50.5)	278	138.7 (49.9)
45	60	74	40.3 (54.5)	100	53.2 (53.2)	124	64.9 (52.3)
45	65	42	24.2 (57.6)	56	31.3 (55.9)	69	37.8 (54.9)
45	70	27	16.4 (60.7)	36	21.1 (58.6)	44	25.2 (57.3)
45	75	19	12.1 (63.8)	24	14.8 (61.7)	29	17.5 (60.2)
50	60	162	91.5 (56.5)	220	122.2 (55.5)	275	151.5 (55.0)
50	65	73	43.5 (59.6)	98	57.1 (58.3)	121	69.5 (57.5)
50	70	41	25.8 (62.8)	55	33.6 (61.1)	67	40.2 (60.0)
50	75	27	17.8 (65.8)	34	21.8 (64.1)	42	26.3 (62.7)
55	65	159	97.8 (61.5)	214	129.7 (60.6)	267	160.2 (60.0)
55	70	71	45.9 (64.7)	94	59.6 (63.4)	117	73.2 (62.6)
55	75	40	27.2 (67.9)	52	34.5 (66.3)	64	41.7 (65.2)
60	70	152	101.1 (66.5)	204	133.9 (65.6)	253	164.6 (65.1)
60	75	68	47.4 (69.8)	89	61.0 (68.5)	109	73.8 (67.7)
60	80	38	27.8 (73.1)	48	34.4 (71.6)	59	41.6 (70.5)
65	75	142	101.6 (71.6)	189	133.6 (70.7)	234	164.1 (70.1)
65	80	63	47.2 (74.9)	81	59.7 (73.7)	99	72.2 (72.9)
65	85	34	26.7 (78.5)	44	33.8 (76.8)	52	39.5 (75.9)
70	80	129	98.9 (76.6)	170	128.8 (75.8)	209	157.2 (75.2)
70	85	56	44.8 (80.1)	72	56.8 (78.9)	87	67.9 (78.1)
75	85	113	92.3 (81.7)	147	118.9 (80.9)	179	143.8 (80.3)
75	90	48	40.9 (85.3)	60	50.5 (84.2)	71	59.3 (83.5)
80	90	93	80.7 (86.8)	119	102.4 (86.0)	143	122.3 (85.5)

*R*_*UL*_ upper limit of RED zone (expressed as percentage of total sample), *G*_*LL*_ lower limit of GREEN zone (expressed as percentage of total sample), *A*_*C*_ AMBER-statistical significance threshold (within the AMBER zone) where an observed estimate below the cut-point will result in a non-significant result (*p* ≥ 0.05) and figures at or above the cut-point will be significant (*p* < 0.05) (%, as a percentage of *n*)

Sample sizes were derived using the normal approximation to the binomial distribution (with continuity correction) formula given in the Appendix, which by convention is stable for *np* > 5 and *n*(1 − *p*) > 5.

For this approach, *A*_*C*_% is calculated from the 1-sided upper 95% confidence limit for the null proportion: 100% × (*R*_*UL*_ + *z*_*1−α*_√((*R*_*UL*_(1 − *R*_*UL*_))/*n*)) [e.g. for *R*_*UL*_ = 20% v *G*_*LL*_ = 35%, *n* = 78, power 90%: *A*_*C*_% = 100% × (0.2 + 1.645√((0.2(1 − 0.2))/78)) = 27.5%. In the example this is expressed as a proportion (0.275)]

The *A*_*C*_ values do not account for the continuity correction (− 0.5 deduction) which would need to be applied to the observed count from a study prior to cross-checking against the *A*_*C*_ cut-offs provided here

**Table 2:** Sample size and significance cut-points for (G_LL_-R_UL_) differences for a one-sample test, power (80%, 90%, 95%) and 1-tailed 5% significance level based on the binomial exact test

***R***_***UL***_	***G***_***LL***_	***α*** (0.05), ***β*** (0.2)		***α*** (0.05), ***β*** (0.1)		***α*** (0.05), ***β*** (0.05)	
%	%	***n***	***A***_***c***_ (%)	***n***	***A***_***c***_ (%)	***n***	***A***_***c***_ (%)
10	20	78	13 (16.7)	109	17 (15.6)	135	20 (14.8)
15	25	101	22 (21.8)	136	28 (20.6)	176	35 (19.9)
15	30	48	12 (25.0)	64	15 (23.4)	85	19 (22.4)
20	30	116	31 (26.7)	160	41 (25.6)	204	51 (25.0)
20	35	56	17 (30.4)	77	22 (28.6)	98	27 (27.6)
20	40	35	12 (34.3)	47	15 (31.9)	60	18 (30.0)
25	35	129	41 (31.8)	179	55 (30.7)	230	69 (30.0)
25	40	62	22 (35.5)	83	28 (33.7)	107	35 (32.7)
25	45	36	14 (38.9)	49	18 (36.7)	62	22 (35.5)
25	50	26	11 (42.3)	33	13 (39.4)	42	16 (38.1)
30	40	144	53 (36.8)	193	69 (35.8)	248	87 (35.1)
30	45	67	27 (40.3)	93	36 (38.7)	114	43 (37.7)
30	50	39	17 (43.6)	53	22 (41.5)	67	27 (40.3)
30	55	25	12 (48.0)	36	16 (44.4)	44	19 (43.2)
30	60	17	9 (52.9)	25	12 (48.0)	28	13 (46.4)
35	45	148	62 (41.9)	206	84 (40.8)	262	105 (40.1)
35	50	68	31 (45.6)	96	42 (43.8)	119	51 (42.9)
35	55	41	20 (48.8)	53	25 (47.2)	68	31 (45.6)
35	60	26	14 (53.8)	36	18 (50.0)	45	22 (48.9)
35	65	19	11 (57.9)	24	13 (54.2)	29	15 (51.7)
40	50	158	74 (46.8)	214	98 (45.8)	268	121 (45.1)
40	55	71	36 (50.7)	94	46 (48.9)	119	57 (47.9)
40	60	42	23 (54.8)	56	29 (51.8)	67	34 (50.7)
40	65	28	16 (57.1)	34	19 (55.9)	45	24 (53.3)
40	70	19	12 (63.2)	25	15 (60.0)	28	16 (57.1)
45	55	154	80 (51.9)	220	112 (50.9)	269	135 (50.2)
45	60	70	39 (55.7)	98	53 (54.1)	119	63 (52.9)
45	65	42	25 (59.5)	54	31 (57.4)	68	38 (55.9)
45	70	25	16 (64.0)	36	22 (61.1)	44	26 (59.1)
45	75	16	11 (68.8)	23	15 (65.2)	29	18 (62.1)
50	60	158	90 (57.0)	213	119 (55.9)	268	148 (55.2)
50	65	69	42 (60.9)	93	55 (59.1)	119	69 (58.0)
50	70	37	24 (64.9)	53	33 (62.3)	67	41 (61.2)
50	75	23	16 (69.6)	33	22 (66.7)	42	27 (64.3)
55	65	150	93 (62.0)	210	128 (61.0)	262	158 (60.3)
55	70	70	46 (65.7)	92	59 (64.1)	114	72 (63.2)
55	75	37	26 (70.3)	50	34 (68.0)	62	41 (66.1)
60	70	143	96 (67.1)	197	130 (66.0)	248	162 (65.3)
60	75	62	44 (71.0)	85	59 (69.4)	107	73 (68.2)
60	80	36	27 (75.0)	45	33 (73.3)	60	43 (71.7)
65	75	133	96 (72.2)	180	128 (71.1)	230	162 (70.4)
65	80	55	42 (76.4)	75	56 (74.7)	98	72 (73.5)
65	85	31	25 (80.6)	42	33 (78.6)	52	40 (76.9)
70	80	119	92 (77.3)	164	125 (76.2)	204	154 (75.5)
70	85	49	40 (81.6)	69	55 (79.7)	85	67 (78.8)
75	85	103	85 (82.5)	139	113 (81.3)	176	142 (80.7)
75	90	45	39 (86.7)	55	47 (85.5)	70	59 (84.3)
80	90	82	72 (87.8)	112	97 (86.6)	135	116 (85.9)

*R*_*UL*_ upper limit of RED zone (expressed as percentage of total sample), *G*_*LL*_ lower limit of GREEN zone (expressed as percentage of total sample), *A*_*C*_ AMBER-statistical significance threshold (within the AMBER zone) where an observed estimate below the cut-point will result in a non-significant result (*p* ≥ 0.05) and figures at or above the cut-point will be significant (*p* < 0.05) (%, expressed as a percentage of sample size (*n*))

### Multi-criteria assessment

We recommend that progression for all key feasibility criteria should be considered separately, and hence overall progression would be determined by the worst-performing criterion, e.g. RED if at least one signal is RED, AMBER if none of the signals fall into RED but at least one falls into AMBER and GREEN if all signals fall into the GREEN zone. Hence, the GREEN signal to ‘GO’ across the set of individual criteria will give indication that progression to a main trial can take place without any necessary changes. A signal to ‘STOP’ and not proceed to a main trial is recommended if any of the observed estimates are ‘unacceptably’ low (i.e. fall within the RED zone). Otherwise, where neither ‘GO’ nor ‘STOP’ are signalled, the design of the trial will need amending by indication of subpar performance on one or more of the criteria.

Sample size requirements across multi-criteria will vary according to the designated parameters linked to the progression criteria, which may be set at different stages of the study on different numbers of patients (e.g. those screened, eligible, recruited and randomised, allocated to the intervention arm, total followed up). The overall size needed will be dictated by the requirement to power each of the multi-criteria statistical tests. Since these tests will yield separate conclusions in regard to the decision to ‘STOP’, ‘AMEND’ or ‘GO’ across all individual feasibility criteria there is no need to consider a multiple testing correction with respect to alpha. However, researchers may wish to increase power (and hence, sample size) to ensure adequate power to detect ‘GO’ signals across the collective set of feasibility criteria. For example, powering at 90% across three criteria (assumed independent) will ensure a collective power of 73% (i.e. 0.9^3^), which may be considered reasonable, but 80% power across five criteria will reduce the power of the combined test to 33%. The final three columns of Table 1 cover the sample sizes required for 95% power, which may address collective multi-criteria assessment when considering keeping a high overall statistical power.

### Further expansion of AMBER zone

Within the same sample size framework, the AMBER zone may be further split to indicate whether ‘minor’ or ‘major’ amendments are required according to the significance of the *p* value. Consider a 2-way split in the AMBER zone denoted by cut-off *A*_*C*_, which indicates the threshold for statistical significance, where an observed estimate below the cut-point will result in a non-significant result and an estimate at or above the cut-point a significant result. Let AMBER_R_ denote the region of Amber zone adjacent to the RED zone between *R*_*UL*_ and *A*_*C*_, and AMBER_G_ denote the region of AMBER zone between *A*_*C*_ and *G*_*LL*_ adjacent to the GREEN zone. This would draw on two possible levels of amendment (‘major’ AMEND and ‘minor’ AMEND) and the re-configured approach would follow as:

- *E* ≤ *R*_*UL*_ [*P* value non-significant (*P ≥ α*)] -> RED (unacceptable—STOP)
- *R*_*UL*_ < *E* < *G*_*LL*_ -> AMBER (potentially acceptable—AMEND)
  - *R*_*UL*_ *< E < G*_*LL*_ *and P ≥ α* {*R*_*UL*_ *< E < A*_*c*_} -> AMBER_R_ (major AMEND)
  - *R*_*UL*_ *< E < G*_*LL*_ *and P < α* { *A*_*c*_ *≤ E < G*_*LL*_} -> AMBER_G_ (minor AMEND)
- *E* ≥ *G*_*LL*_ [*P* value significant (*P < α*)] -> GREEN (acceptable—GO)

In Tables 1 and 2 in relation to designated sample sizes for different *R*_*UL*_ and *G*_*LL*_ and specified *α* and β, we show the corresponding cut-points for statistical significance (*p* < 0.05) both in absolute terms of sample number (*n*) [*A*_*C*_] and as a percentage of the total sample sizes [*A*_*C*_*%*].

## Results

A motivating example (aligned to the normal approximation approach) is presented in Table 3, which illustrates a pilot trial with three progression criteria. Table 4 presents the sample size calculations for the example scenario following the 3-tiered approach, and Table 5 gives the sample size calculations for the example scenario using the extended 4-tiered approach. Cut-points for the feasibility outcomes relating to the shown sample sizes are also presented to show RED, AMBER and GREEN zones for each of the three progression criteria.

**Table 3:** Motivating example—feasibility trial for oral protein energy supplements as flavoured drinks to improve nutritional status in children with cystic fibrosis

A feasibility trial is being set up to see whether children aged 2 to 15 years with cystic fibrosis will take oral protein energy supplements as flavoured drinks to improve their nutritional status, compared to receiving dietary advice alone. Children are to be randomised in a 1:1 allocation ratio using a parallel two-arm design. The research team wants to be sure they can meet three feasibility objectives before they go ahead and plan the main trial: reasonable recruitment uptake, high treatment fidelity (i.e. extent to which dietician practitioners comply with the treatment protocol) and adequate retention of children at follow up. The team asks their senior statistician to help them decide on an appropriate methodology including pilot sample size. The statistician suggests a traffic light approach incorporating hypothesis testing of the feasibility outcomes. Together, the team devise three progression criteria that should be met before the main trial can be considered feasible as follows: a. At least 35% of the children screened as eligible should be recruited but the trial will not be feasible if recruitment uptake is 20% or less. b. A high level of treatment fidelity should be maintained with 75% or more children being given the correct treatment plan by the dietician, but if 50% or less children are given the plan as specified in the protocol then the trial is not feasible. c. 85% or more of the children should be retained in the study at follow up, with 65% or less retention indicating that the main trial is not feasible. The decision criteria and required sample size around these are detailed through two possible approaches within Table 4 (simple 3-tier approach) and Table 5 (extended 4-tier approach). The statistician is to use the normal approximation method (with continuity correction) for the sample size calculation and analysis.

**Table 4:** Case illustration (standard 3-tiered approach)

A two-arm parallel design (1:1 allocation to intervention and control arms) with three key feasibility objectives, to assess (i) recruitment uptake (percent of screened patients recruited), (ii) treatment fidelity and (iii) participant retention (follow up). Hypothesis testing incorporates *α* (1-sided) = 5% and power = 90%. The normal approximation method is used. Assume the progression criteria (and affiliated sample size requirements) for each are as follows: (i) Recruitment uptake ≤ 20% (RED zone) and ≥ 35% (GREEN zone) {*R*_*UL*_ = 20%, *G*_*LL*_ = 35%} → Required sample size *n* = 78 [total screened patients] (ii) Treatment fidelity ≤ 50% (RED zone) and ≥ 75% (GREEN zone) {*R*_*UL*_ = 50%, *G*_*LL*_ = 75%} → Required sample size *n* = 34 [intervention arm only] (iii) Follow up: ≤ 65% (RED zone), ≥ 85% (GREEN zone) {*R*_*UL*_ = 65%, *G*_*LL*_ = 85%} → Required sample size *n* = 44 (total randomised participants with 22 per arm) The sample sizes across criteria (i)-(iii) are at different levels—(i) is at the level of screened patients, whereas (ii)–(iii) are at the level of randomised patients. To meet criteria (i), we need *n*_*s*_ ≥ 78 (although we will recruit *n*_*s*_ = 200 (i.e. (1/0.35) × *n*_*r*_ (rounded up to 200)) where 0.35 is the expected proportion uptake of the total number screened), and for (ii)–(iii), we need *n*_*r*_ = 68 (34 per arm, based on (ii)). Taking each of the objectives in turn (and the updated sample sizes to meet the multi-criteria objectives), we express progression criteria for the three objectives as follows: (i) Recruitment uptake [required *n*_*s*_ ≥ 78; expected *n*_*s*_ = 200; maximum *n*_*s*_ = 340 (i.e. (1/0.2)x *n*_*r*_)] • *E* ≤ 0.2 [*P* ≥ 0.05] -> RED (STOP) • 0.2 < *E* < 0.35 -> AMBER (AMEND) • *E* ≥ 0.35 [P < 0.05] -> GREEN (GO) Signals for expected *n*_*s*_ = 200: 0 to 40 (RED), > 40 to < 70 (AMBER) and 70 to 200 (GREEN) {i.e. 0.2 × 200 = 40; 0.35 × 200 = 70} (ii) Treatment fidelity [*n*_*i*_ = 34 (intervention arm only)] • *E* ≤ 0.5 [*P* ≥ 0.05] -> RED (STOP) • 0.5 < *E* < 0.75 -> AMBER (AMEND) • *E* ≥ 0.75 [*P* < 0.05] -> GREEN (GO) Signals for *n*_*i*_ = 34: 0 to 17 (RED), > 17 to < 25.5 (AMBER) and 25.5 to 34 (GREEN) {i.e. 0.5 × 34 = 17; 0.75 × 34 = 25.5} (iii) Follow up [*n*_*r*_ = 68 (intervention and control arms)] • *E* ≤ 0.65 [*P* ≥ 0.05] -> RED (STOP) • 0.65 < *E* < 0.85 -> AMBER (AMEND) • *E* ≥ 0.85 [*P* < 0.05] -> GREEN (GO) Signals for *n*_*r*_ = 68: 0 to 44.2 (RED), > 44.2 to < 57.8 (AMBER) and 57.8 to 68 (GREEN) {i.e. 0.65 × 68 = 44.2; 0.85 × 68 = 57.8} [Note: The continuity correction (− 0.5 deduction) needs to be applied to the observed count from the study for each criterion prior to assessing into which signal band it falls] In accordance with the multi-criteria aim, the decision to proceed would be based on the worst signal ➢ If signal = RED for (i) or (ii) or (iii) -> overall signal is RED ➢ Else, if no signal is RED but signal = AMBER for (i) or (ii) or (iii) -> overall signal is AMBER ➢ Else, if signals = GREEN for (i) and (ii) and (iii) -> overall signal is GREEN	

*R*_*UL*_ upper limit of RED zone, *G*_*LL*_ lower limit of GREEN zone, *n*_*s*_ number of screened patients who are eligible to being randomised, *n*_*r*_ number of eligible patients randomised, *n*_*i*_ number of patients randomised to the intervention arm

**Table 5:** Case illustration (re-visited using 4-tiered approach)

Taking each of the objectives in turn, we re-express the progression criteria for the three objectives according to the 4-tiered approach, as follows: (i) Recruitment uptake [expected *n*_*s*_ = 200] • *E* ≤ 0.2 [*P* ≥ 0.05] -> RED (STOP) • 0.2 < *E* < 0.35 -> AMBER (AMEND)//{*A*_*c*_ = 0.247 (i.e. 0.2 + 1.645√(0.2 × 0.8/200))}* o 0.2 < *E* < 0.247 [*P* ≥ 0.05] -> AMBER_R_ (AMEND-major) o 0.247 ≤ *E* < 0.35 [*P* < 0.05] -> AMBER_G_ (AMEND-minor) • *E* ≥ 0.35 [*P* < 0.05] -> GREEN (GO) Signals for *n*_*s*_ = 200: 0 to 40 (RED), > 40 to < 49.4 (AMBER_R_), 49.4 to < 70 (AMBER_G_) and 70 to 200 (GREEN) {i.e. 0.2 × 200 = 40, 0.247 × 200 = 49.4, 0.35 × 200 = 70} (ii) Treatment fidelity [*n*_*i*_ = 34 (intervention arm only)] • *E* ≤ 0.5 [*P* ≥ 0.05] -> RED (STOP) • 0.5 < *E* < 0.75 -> AMBER (AMEND)//{*A*_*C*_ = 0.641 (i.e. 0.5 + 1.645√(0.5 × 0.5/34))—as shown in Table 1}* o 0.5 < *E* < 0.641 [*P* ≥ 0.05] -> AMBER_R_ (AMEND-major) o 0.641 ≤ *E* < 0.75 [*P* < 0.05] -> AMBER_G_ (AMEND-minor) • *E* ≥ 0.75 [*P* < 0.05] -> GREEN (GO) Signals for n_i_ = 34: 0 to 17 (RED), > 17 to < 21.79 (AMBER_R_), 21.79 to < 25.5 (AMBER_G_) and 25.5 to 34 (GREEN) {i.e. 0.5 × 34 = 17, 0.641 × 34 = 21.794, 0.75 × 34 = 25.5} (iii) Follow up [*n*_*r*_ = 68 (intervention and control arms)] • *E* ≤ 0.65 [*P* ≥ 0.05] -> RED (STOP) • 0.65 < *E* < 0.85 -> AMBER (AMEND)//{*A*_*c*_ = 0.745 (i.e. 0.65 + 1.645x√(0.65 × 0.35/68))}* o 0.65 < *E* < 0.745 [*P* ≥ 0.05] -> AMBER_R_ (AMEND-major) o 0.745 ≤ *E* < 0.85 [*P* < 0.05] -> AMBER_G_ (AMEND-minor) • *E* ≥ 0.85 [*P* < 0.05] -> GREEN (GO) Signals for *n*_*r*_ = 68: 0 to 44.2 (RED), > 44.2 to < 50.66 (AMBER_R_), 50.66 to < 57.8 (AMBER_G_) and 57.8 to 70 (GREEN) {i.e. 0.65 × 68 = 44.2, 0.745 × 68 = 50.66, 0.85 × 68 = 57.8} [Note: The continuity correction (-0.5 deduction) needs to be applied to the observed count from the study for each criterion prior to assessing into which signal band it falls] In accordance with the multi-criteria aim, the decision to proceed would be based on the worst signal (as in Table 4)	

*n*_*s*_ number of screened patients who are eligible to being randomised, *n*_*r*_ number of eligible patients randomised, *n*_*i*_ number of patients randomised to the intervention arm

*A_*C*_ is calculated from the 1-sided upper 95% confidence limit for the null proportion: *R*_*UL*_ + *z*_*1−α*_√((*R*_*UL*_(1 − *R*_*UL*_))/*n*) where *z*_*1−α*_ = 1.645 (for 1-sided 5% significance test)

Overall sample size requirement should be dictated by the multi-criteria approach. This is illustrated in Table 4 where we have three progression criteria each with a different denominator population. For recruitment uptake, the denominator denotes the total number of children screened and the numerator the number of children randomised; for follow-up, the denominator is the number of children randomised with the numerator being number of those randomised who are successfully followed up; and lastly for treatment fidelity, the denominator is the number allocated to the intervention arm with the numerator being the number of children who were administered the treatment correctly by the dietician. In the example in order to meet the individual ≥ 90% power requirement for all three criteria we would need: (i) for recruitment, the number to be screened to be 78; (ii) for treatment fidelity, the number in the intervention arm to be 34; and (iii) for follow up, the number randomised to be 44. In order to determine the overall sample size for the whole study, we base our decision on the criterion that requires the largest numbers, which is the treatment fidelity criterion which requires 68 to be randomised. We cannot base our decision on the 78 required to be screened for recruitment because this would give only an expected number of 28 randomised (i.e. 35% of 78). If we expect 35% recruitment uptake, then we need to inflate the total 68 (randomised) to be 195 (1/0.35 × 68) children to be screened (rounded to 200). This would give 99.9%, 90% and 98.8% power for criteria (i), (ii) and (iii), respectively (assuming 68 of the 200 screened are randomised), giving a very reasonable collective 88.8% power of rejecting the null hypotheses over the three criteria if the alternative hypotheses (for acceptable feasibility outcomes) are true in each case.

**Fig. 1:**
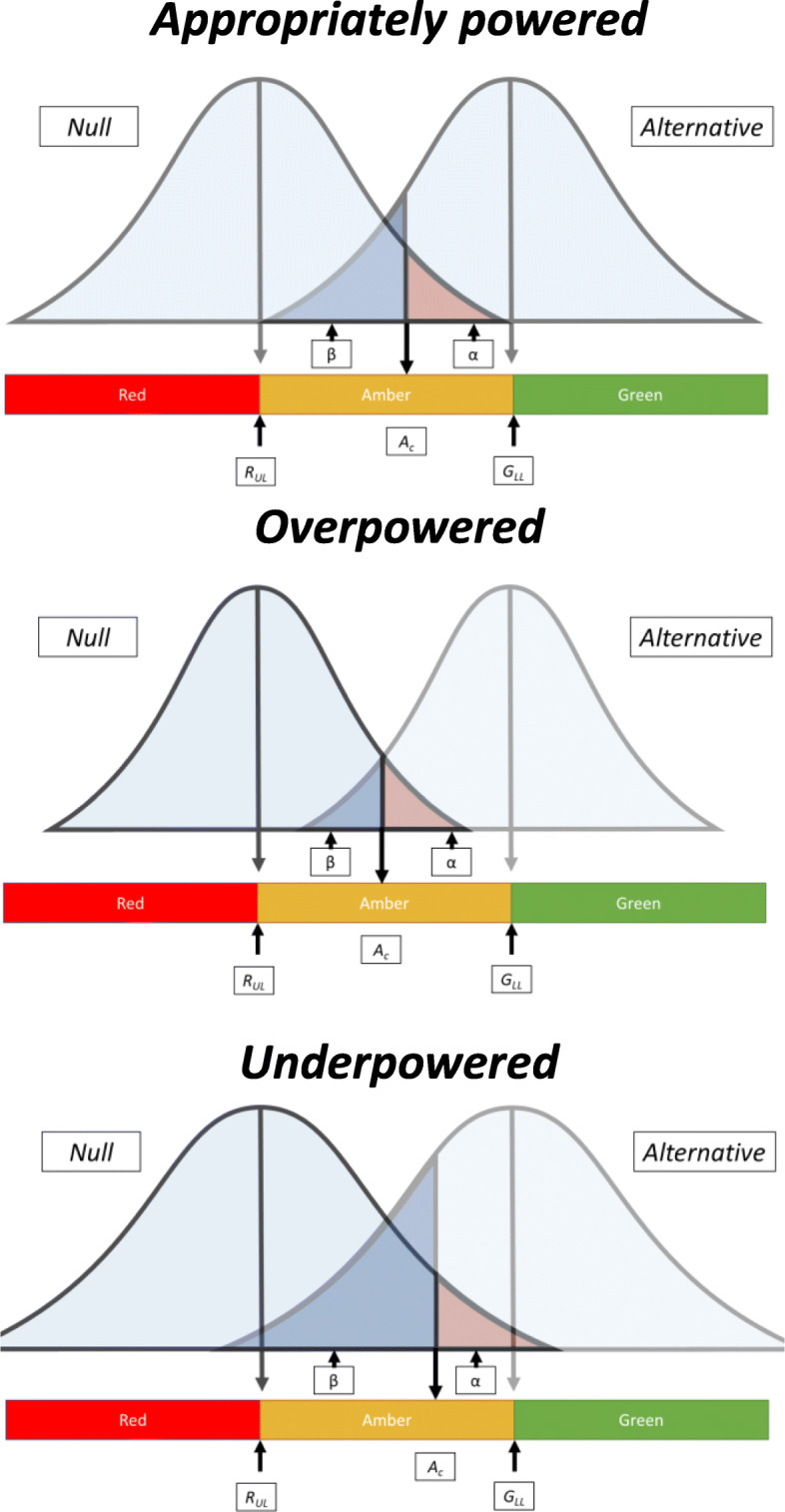
Illustration of power using the 1-tailed hypothesis testing against the traffic light signalling approach to pilot progression. *E*, observed point estimate; *R*_*UL*_, upper limit of RED zone; *G*_*LL*_, lower limit of GREEN zone; *Ac*, cut-off for statistical significance (at the 1-sided 5% level); *α*, type I error; *β*, type II error

Inherent in our approach are the probabilities around sample size, power and hypothesised feasibility parameters. For example, taking the cut-offs from treatment fidelity as a feasibility outcome from Table 4 (ii), we set a lower GREEN zone limit of *G*_*LL*_ = 0.75 (“acceptable” (hypothesised alternative value)) and an upper RED zone limit of *R*_*UL*_ = 0.5 (“not acceptable” (hypothesised null value)) for rejecting the null for this criterion based on 90% power and a 1-sided 5% significance level (alpha). Figure 1 presents the normal probability density functions for *ε*, for the null and alternative hypotheses. In the illustration this would imply through normal sampling theory that if *G*_*LL*_ holds true (i.e. true recruitment uptake (*ε*) = *G*_*LL*_) there would be the following:

- A probability of 0.1 (type II error probability β) of the estimate falling within RED/AMBER_R_ zones (i.e. blue shaded area under the curve to the left of *A*_*C*_ where the test result will be non-significant (*p* ≥ 0.05))
- Probability of 0.4 of it falling in the AMBER_G_ zone (i.e. area under the curve to the right of *A*_*C*_ but below *G*_*LL*_)
- Probability of 0.5 of the estimate falling in the GREEN zone (i.e. *G*_*LL*_ and above).

If *R*_*UL*_ (the null) holds true (i.e. true feasibility outcome (*ε*) = *R*_*UL*_), there would be the following:

- A probability of 0.05 (one-tailed type I error probability *α*) of the statistic/estimate falling in the AMBER_G_/GREEN zones (i.e. pink shaded area under the curve to the right of *A*_*C*_ where the test result will be significant (*p* < 0.05) as shown within Fig. 1)
- Probability of 0.45 of it falling in the AMBER_R_ zone (i.e. to the left of *A*_*C*_ but above *R*_*UL*_)
- Probability of 0.5 of the estimate falling in the RED zone (i.e. *R*_*UL*_ and below)

Figure 1 also illustrates how changing the sample size affects the sampling distribution and power of the analysis around the set null value (at *R*_*UL*_) when the hypothesised alternative (*G*_*LL*_) is true. The figure emphasises the need for a large enough sample to safeguard against under-powering of the pilot analysis (as shown in the last plot which has a wider bell-shape than the first two plots and where the size of the beta probability is increased).

**Fig. 2:**
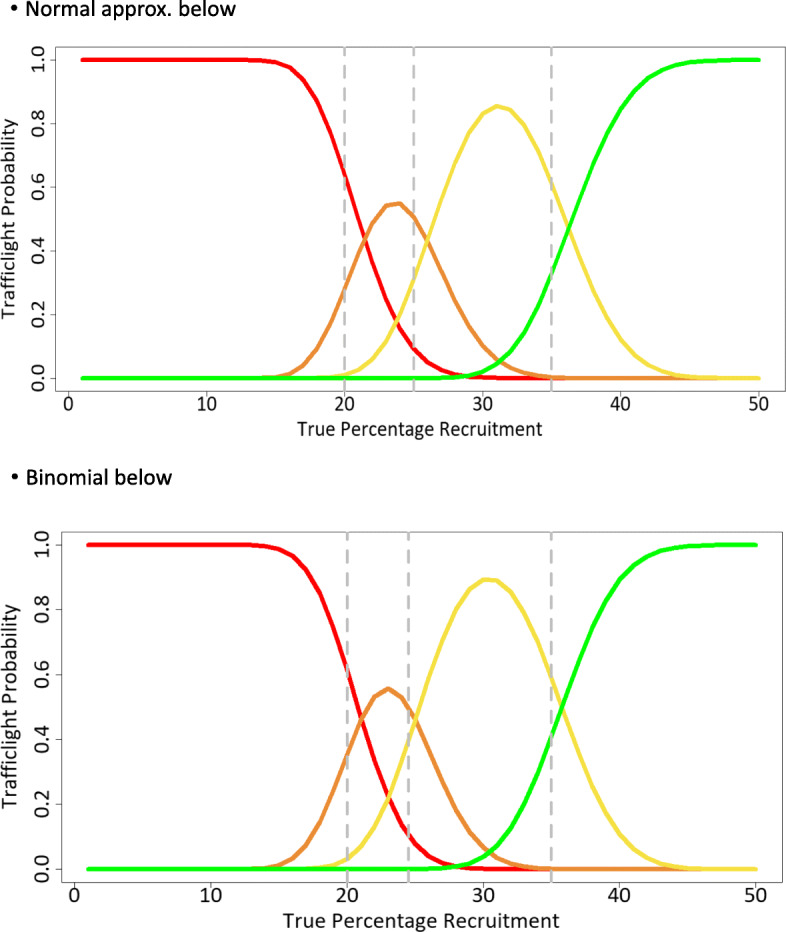
Probability of traffic light given true underlying probability of an event using the example from Table 5 (i). Two plots are presented: **a** relating to normal approximation approach and **b** relating to binomial exact approach. Based on *n* = 200, *R*_*UL*_ = 40 and *G*_*LL*_ = 70

Figure 2 plots the probabilities of making each type of traffic light decision as functions of the true parameter value (focused on the recruitment uptake example from Table 5 (i)). Additional file 1 presents the *R* code for reproducing these probabilities and enables readers to insert different parameter values.

## Discussion

The methodology introduced in this article provides an innovative formal framework and approach to sample size derivation, aligning sample size requirement to progression criteria with the intention of providing greater transparency to the progression process and full engagement with the standard aims and objectives of pilot/feasibility studies. Through the use of both alpha and beta parameters (rather than alpha alone), the method ensures rigour and capacity to address the progression criteria by ensuring there is adequate power to detect an acceptable threshold for moving forward to the main trial. As several key process outcomes are assessed in parallel and in combination, the method embraces a composite multi-criterion approach that appraises signals for progression across all the targeted feasibility measures. The methodology extends beyond the requirement for ‘sample size justification but not necessarily sample size calculation’ [28].

The focus of the strategy reported here is on process outcomes, which align with the recommended key objectives of primary feasibility evaluation for pilot and feasibility studies [2, 24] and necessary targets to address key issues of uncertainty [29]. The concept of justifying progression is key. Charlesworth et al. [30] developed a checklist for intended use in decision-making on whether pilot data could be carried forward to a main trial. Our approach builds on this philosophy by introducing a formalised hypothesis test approach to address the key objectives and pilot sample size. Though the suggested sample size derivation focuses around the key process objectives, it may also be the case that other objectives are also important, e.g. assessment of precision of clinical outcome parameters. In this case, researchers may also wish to ensure that the size of the study suitably covers the needs of those evaluations, e.g. to estimate the SD of the intended clinical outcome, then the overall sample size may be boosted to cover this additional objective [10]. This tallies with the review by Blatch-Jones et al. [31] who reported that testing recruitment, determining the sample size and numbers available, and the intervention feasibility were the most commonly used targets of pilot evaluations.

Hypothesis testing in pilot studies, particularly in the context of effectiveness/efficacy of clinical outcomes, has been widely criticised due to the improper purpose and lack of statistical power of such evaluations [2, 20, 21, 23]. Hence, pilot evaluations of clinical outcomes are not expected to include hypothesis testing. Since the main focus is on feasibility the scope of the testing reported here is different and importantly relates back to the recommended objectives of the study whilst also aligning with nominated progression criteria [2]. Hence, there is clear justification for this approach. Further, for the simple 3-tiered approach hypothesis testing is somewhat hypothetical: there is no need to physically carry out a test since the zonal positioning of the observed sample statistic estimate for the feasibility outcome will determine the decision in regard to progression; thus adding to the simplicity of the approach.

The link between the sample size and need to adequately power the study to detect a meaningful feasibility outcome gives this approach the extra rigour over the confidence interval approach. It is this sample size-power linkage that is key to the determination of the respective probabilities of falling into the different zones and is a fundamental underpinning to the methodological approach. In the same way as for a key clinical outcome in a main trial where the emphasis is not just on alpha but also on beta thereby addressing the capacity to detect a clinically significant difference, similarly, our approach is to ensure there is sufficient capacity to detect a meaningful signal for progression to a main trial if it truly exists. A statistically significant finding in this context will at least provide evidence to reject RED (signifying a decision to STOP) and in the 4-tiered case it would fall above AMBER_R_ (decision to major-AMEND); hence, the estimate will fall into AMBER_G_ or GREEN (signifying a decision to minor-AMEND or GO, respectively). The importance of adequately powering the pilot trial to address a feasibility criterion can be simply illustrated. For example, if we take *R*_*UL*_ as 50% and *G*_*LL*_ as 75% but with two different sample sizes of *n* = 25 and *n* = 50; the former would have 77.5% power of rejecting RED on the basis of a 1-sided 5% alpha level whereas the larger sample size would have 97.8% power of rejecting RED. So, if *G*_*LL*_ holds true, there would be 20% higher probability of rejecting the null and being in the AMBER_G_/GREEN zone for the larger sample giving an increased chance of progressing to the main trial. It will be necessary to carry out the hypothesis test for the extended 4-tier approach if the observed statistic (*E*) falls in the AMBER zone to determine statistical significance or not, which will inform whether the result falls into the ‘minor’ or ‘major’ AMBER sub-zones.

We provide recommended sample sizes within a look-up grid relating to perceived likely progression cut-points to aid quick access and retrievable sample sizes for researchers. For a likely set difference in proportions between hypothesised null and alternative parameters of 0.15 to 0.25 when *α* = 0.05 and β = 0.1 the corresponding total sample size requirements for the approach of normal approximation with continuity correction take the range of 33 to 100 (median 56) [similarly these are 33–98 (median 54) for the binomial exact method]. Note, for treatment fidelity/adherence/compliance particularly, the marginal difference could be higher, e.g. ≥ 25%, since in most situations we would anticipate and hope to attain a high value for the outcome whilst being prepared to make necessary changes within a wide interval of below par values (and providing the value is not unacceptably low). As this relates to an arm-specific objective (relating to evaluation of the intervention only), then a usual 1:1 pilot will require twice the size; hence, the arm-specific sample size powered for detecting a ≥ 25% difference from the null would be about 34 (or lower)—as depicted from our illustration (Table 4 (ii), equating to *n* ≤ 68 overall for a 1:1 pilot; intervention and control arms). Hence, we expect that typical pilot sizes of around 30–40 randomised per arm [16] would likely fit with the proposed methodology within this manuscript (the number needed for screening being extrapolated upward of this figure) but if a smaller marginal difference (e.g. ≤ 15%) is to be tested then these sample sizes may fall short. We stress that the overall required sample size needs to be carefully considered and determined in line with the hypothesis testing approach across all criteria ensuring sufficiently high power. In our paper, we have made recommendations regarding various sample sizes based on both the normal approximation (with continuity correction) and binomial exact approaches; these are conservative compared to the Normal approximation (without continuity correction).

Importantly, the methodology outlines the necessary multi-criterion approach to the evaluation of pilot and feasibility studies. If all progression criteria are performing as well as anticipated (highlighting ‘GO’ according to all criteria), then the recommendation of the pilot/feasibility study is that all criteria meet their desired levels with no need for adjustment and the main trial can proceed without amendment. However, if the worst signal (across all measured criteria) is an AMBER signal, then adjustment will be required against those criteria that fall within that signal. Consequently, there is the possibility that the criteria may need subsequent re-assessment to re-evaluate processes in line with updated performance for the criteria in question. If one or more of the feasibility statistics fall within the RED zone then this signals ‘STOP’ and concludes that a main trial is not feasible based on those criteria. This approach to collectively appraising progression based on the results of all feasibility outcomes assessed against their criteria will be conservative as the power of the collective will be lower than the individual power of the separate tests; hence, it is recommended that the power of the individual tests is set high enough (for example, 90–95%) to ensure the collective power is high enough (e.g. at least 70 or 80%) to detect true ‘GO’ signals across all the feasibility criteria.

In this article, we also expand the possibilities for progression criterion and hypothesis testing where the AMBER zone is sub-divided arbitrarily based on the significance of the *p* value. This may work well when the AMBER zone has a wide range and is intended to provide a useful and workable indication of the level of amendment (‘minor’ (non-substantive) or ‘major’ (substantive)) required to progress to the main trial. Examples of substantial amendments include study re-design with possible re-appraisal and change of statistical parameters, inclusion of several additional sites, adding further data recruitment methods, significant reconfiguration of exclusions, major change to the method of delivery of trial intervention to ensure enhanced treatment fidelity/adherence, enhanced measures to systematically ensure greater patient compliance with allocated treatment, additional mode(s) of collecting and retrieving data (e.g. use of electronic data collection methods in addition to postal questionnaires). Minor amendments include small changes to the protocol and methodology, e.g. addition of one or two sites for attaining a slightly higher recruitment rate, use of occasional reminders in regard to treatment protocol and adding a further reminder process for boosting follow up. For the most likely parametrisation of *α* = 0.05/β = 0.1, the AMBER zone division will be roughly at the midpoint. However, researchers can choose this point (the major/minor cut-point) based on decisive arguments around how major and minor amendments would align to the outcome in question. This should be factored within the process of sample size determination for the pilot. In this regard, a smaller sample size will move *A*_*C*_ upwards (due to increased standard error/reduced precision) and hence increase the size of the AMBER_R_ zone in relation to AMBER_G_ (whereas a larger sample size will shift *A*_*C*_ downwards and do the opposite, increasing the ratio of AMBER_G_:AMBER_R_). From Table 1, for smaller sample sizes (related to 80% power) the AMBER_R_ zone makes up 56–69% of the total amber zone across presented scenarios, whereas this falls to 47–61% for samples (related to 90% power) and 41–56% for larger samples (related to 95% power) for the same scenarios. Beyond our proposed 4-tier approach, other ways of providing an indication of level of amendment could include evaluation and review of the point and interval estimates or by evaluating posterior probabilities via a Bayesian approach [14, 32].

The methodology illustrated here focuses on feasibility outcomes presented as percentages/proportions, which is likely to be the most common form for progression criteria under consideration. However, the steps that have been introduced can be readily adapted to any feasibility outcomes taking a numerical format, e.g. rate of recruitment per month per centre, count of centres taking part in the study. Also, we point out that in the examples presented in the paper (recruitment, treatment fidelity and percent follow-up), high proportions are acceptable and low ones not. This would not be true for, say, adverse events where a reverse scale is required.

Biased sample estimates are a concern as they may result in a wrong decision being made. This systematic error is over-and-above the possibility of an erroneous decision being made on the basis of sampling error; the latter may be reduced through an increased pilot sample size. Any positive bias will inflate/overestimate the feasibility sample estimate in favour of progressing whereas a negative bias will deflate/underestimate it towards the null and stopping. Both are problematic for opposite reasons; for example, the former may inform researchers that the main trial can ‘GO’ ahead when in fact it will struggle to meet key feasibility targets, whereas the latter may caution against progression when in reality the feasibility targets of a main trial would be met. For example, in regard to the choice of centres (and hence practitioners and participants), a common concern is that the selection of feasibility trial centres might not be a fair and representative sample of the ‘population’ of centres to be used for the main trial. It may be that the host centre (likely used in pilot studies) recruits far better than others (positive bias), thus exaggerating the signal to progress and subsequent recruitment to the main trial. Beets et al. [33] ‘define “risk of generalizability biases” as the degree to which features of the intervention and sample in the pilot study are NOT scalable or generalizable to the next stage of testing in a larger, efficacy/effectiveness trial … whether aspects like who delivers an intervention, to whom it is delivered, or the intensity and duration of the intervention during the pilot study are sustained in the larger, efficacy/effectiveness trial.’ As in other types of studies, safeguards regarding bias should be addressed through appropriate pilot study design and conduct.

Issues relating to progression criteria for internal pilots may be different to those for external pilots and non-randomised feasibility studies. The consequence of a ‘stop’ within an internal pilot may be more serious for stakeholders (researchers, funders, patients) as it would bring an end to the planned continuation into the main trial phase, whereas there would be less at stake for a negative external pilot. By contrast, the consequence of a ‘GO’ signal may work the other way with a clear and immediate gain for the internal pilot whereas for an external pilot, the researchers would still need to apply and get the necessary funding and approvals to undertake an intended main trial. The chances of falling into the different traffic light zones are likely to be quite different between the two designs. Possibly external pilot and feasibility studies are more likely to have estimates falling in and around the RED zone than for internal pilots, reflecting the greater uncertainty in the processes for the former and greater confidence in the mechanisms for trial delivery for the latter. However, to counter this, there are often large challenges with recruitment within internal pilot studies where the target population is usually spread over more diverse sites than may be expected for an external pilot. Despite this possible imbalance, the interpretation of zonal indications remains consistent for external and internal pilot studies. As such, our focus with regard to the recommendations in this article are aligned to requirements for external pilots, though application of this methodology to a degree may similarly hold for internal pilots (and further, to non-randomised studies that can include progression criteria—including longitudinal observational cohorts with the omission of the treatment fidelity criterion).

## Conclusions

We propose a novel framework that provides a paradigm shift towards formally testing feasibility progression criteria in pilot and feasibility studies. The outlined approach ensures rigorous and transparent reporting in line with CONSORT recommendations for evaluation of STOP-AMEND-GO criteria and presents clear progression sign-posting which should help decision-making and inform stakeholders. Targeted progression criteria are focused on recommended pilot and feasibility objectives, particularly recruitment uptake, treatment fidelity and participant retention, and these criteria guide the methodology for sample size derivation and statistical testing. This methodology is intended to provide a more definitive and rounded structure to pilot and feasibility design and evaluation than currently exists. Sample size recommendations will be dependent on the nature and cut-points for multiple key pre-defined progression criteria and should ensure a sufficient sample size for other feasibility outcomes such as review of the precision of clinical parameters to better inform main trial size.

### Supplementary Information

**Additional file 1.** R codes used for Fig. 2.

## Data Availability

Not applicable.

## Appendix

Mathematical formulae for derivation of sample size

The required sample size may be derived using normal approximation to binary response data—using a continuity correction, via Fleiss et al. [26] if the convention of *np* > 5 and *n*(*1 − p*) > 5 holds true:

$$ n={\left(\frac{z_{1-\propto}\sqrt{R_{UL}\left(1-{R}_{UL}\right)+}{z}_{1-\beta}\sqrt{G_{LL}\left(1-{G}_{LL}\right)}}{\left({G}_{LL}-{R}_{UL}\right)}\right)}^2+\frac{1}{\left|{G}_{LL}-{R}_{UL}\right|} $$

where *R*_*UL*_ = upper limit of RED zone; *G*_*LL*_ = lower limit of GREEN zone; *z*_1−*α*_ = one-sided statistical significance level (type I error probability); *z*_1−β_ = beta (type II error probability)

## Abbreviations

Alpha (*α*): Significance level (Type I error probability)
AMBER_G_: AMBER sub-zone split adjacent to the GREEN zone (within 4-tiered approach)
AMBER_R_: AMBER sub-zone split adjacent to the RED zone (within 4-tiered approach)
*A*_*C*_: AMBER-statistical significance threshold (within the AMBER zone) where an observed estimate below the cut-point will result in a non-significant result (p ≥ 0.05) and figures at or above the cut-point will be significant (p < 0.05)
*A*_*C*_%: *A*_*C*_ expressed as a percentage of the sample size
Beta (β): Type II error probability
*E*: Estimate of feasibility outcome
*ε*: True feasibility parameter
*G*_*LL*_: Lower Limit of GREEN zone
*n*: Sample size (n_s_ = number of patients screened; n_r_ = number of patients randomised; n_i_ = number of patients randomised to the intervention arm only)
Power = 1-Beta: (1 – Type II error probability)
*R*_*UL*_: Upper Limit of RED zone
