# Supplementary material for: Determining sample size for progression criteria for pragmatic pilot RCTs: the hypothesis test strikes back!
Source: Pilot Feasibility Stud. 2021 Feb 3;7:40. doi: 10.1186/s40814-021-00770-x (PMC7856754; doi:10.1186/s40814-021-00770-x)
Supplement: Supplementary file 1 — Additional file 1. R codes used for Fig. 2. [file 40814_2021_770_MOESM1_ESM.docx]

**Additional file 1**

Figure 2 presents a plot of the probability of traffic light signals given the true underlying probability of an event, given the following: N=200, R_UL_ = 40 (20%), G_LL_ = 70 (35%). The R codes used for these figures are presented below

a) Normal approximation code:-

install.packages("extrafont")

library(extrafont)

font_import()

loadfonts(device="win")

norm.seq <- 1:200

n = 200

rul = 0.2 ## 40

gll = 0.35 ## 70

ac = min(which(round(pnorm(norm.seq, n*rul, sqrt(n * (rul * (1 - rul)))), 3) > 0.95)) #50

###

vals.seq <- seq(0,1,0.01)

vals.mat <- matrix(NA, nrow = length(vals.seq), ncol = 4)

#####################

plot(pbinom(1:200, 100, prob = 0.5))

plot(pnorm(1:200, n*0.5, sqrt(n*0.5*(1-0.5))))

### If the true probability of a value is P, what is the chance of each traffic light?

vals.mat <- matrix(NA, nrow = length(vals.seq), ncol = 4)

vals.seq <- seq(0,1,0.01)

vals.mat[,1] <- pnorm(40, n*vals.seq, sqrt(n*vals.seq*(1-vals.seq)))

vals.mat[,2] <- -pnorm(41, n*vals.seq, sqrt(n*vals.seq*(1-vals.seq))) + pnorm(50, n*vals.seq, sqrt(n*vals.seq*(1-vals.seq)))

vals.mat[,3] <- -pnorm(51, n*vals.seq, sqrt(n*vals.seq*(1-vals.seq))) + pnorm(70, n*vals.seq, sqrt(n*vals.seq*(1-vals.seq)))

vals.mat[,4] <- 1 - pnorm(71, n*vals.seq, sqrt(n*vals.seq*(1-vals.seq)))

### plot it

par(mar=c(5,6,4,1)+.1)

op <- par(family = "Calibri")

plot(vals.mat[1:50,1], type = "l", col = "red", lwd = 8, ylab = "Trafficlight Proability", xlab = "True Percentage Probability",

cex.lab = 2.5, cex.axis = 2.5)

lines(vals.mat[1:50,2], type = "l", col = "#eb8c34", lwd = 8)

lines(vals.mat[1:50,3], type = "l", col = "#f5e042", lwd = 8)

lines(vals.mat[1:50,4], type = "l", col = "green", lwd = 8)

abline(v = 20, col = "#bfbfbf", lwd = 5, lty = 2)

abline(v = 25, col = "#bfbfbf", lwd = 5, lty = 2)

abline(v = 35, col = "#bfbfbf", lwd = 5, lty = 2)

dev.off()

b) Binomial exact code:-

install.packages("extrafont")

library(extrafont)

font_import()

loadfonts(device="win")

### Set parameters

n = 200

rul = floor(0.2 * n) # 40+

gll = floor(0.35 * n) # 70+

ac = min(which(round(pbinom(1:200, 200, 0.2), 3) > 0.95)) ## 49+

### work out the probabilities

vals.seq <- seq(0,1,0.01)

vals.mat <- matrix(NA, nrow = length(vals.seq), ncol = 4)

for(i in 1:length(vals.seq)){

vals.mat[i,1] <- sum(dbinom(0:39, size = n, vals.seq[i])) ## Red

vals.mat[i,2] <- sum(dbinom(40:48, size = n, vals.seq[i])) ## Amber 1

vals.mat[i,3] <- sum(dbinom(49:69, size = n, vals.seq[i])) ## Amber 2

vals.mat[i,4] <- sum(dbinom(70:200, size = n, vals.seq[i])) ## Green

}

### Plot them

par(mar=c(5,6,4,1)+.1)

op <- par(family = "Calibri")

plot(vals.mat[1:50,1], type = "l", col = "red", lwd = 8, ylab = "Trafficlight Proability", xlab = "True Percentage Probability",

cex.lab = 2.5, cex.axis = 2.5)

lines(vals.mat[1:50,2], type = "l", col = "#eb8c34", lwd = 8)

lines(vals.mat[1:50,3], type = "l", col = "#f5e042", lwd = 8)

lines(vals.mat[1:50,4], type = "l", col = "green", lwd = 8)

abline(v = 20, col = "#bfbfbf", lwd = 5, lty = 2)

abline(v = (49/200) * 100, col = "#bfbfbf", lwd = 5, lty = 2)

abline(v = 35, col = "#bfbfbf", lwd = 5, lty = 2)

dev.off()
